# An examination of Eyal & Hurst’s (2008) framework for promoting retention in resource-poor settings through locally-relevant training: A case study for the University of Guyana Surgical Training Program

**Published:** 2017-04-20

**Authors:** Anupa J. Prashad, Brian H. Cameron, Meghan McConnell, Madan Rambaran, Lawrence E. M. Grierson

**Affiliations:** 1 Health Sciences Education Graduate Program, Faculty of Health Sciences, McMaster University, Ontario, Canada; 2 Department of Surgery, Faculty of Health Sciences, McMaster University, Ontario, Canada; 3 Department of Innovation in Medical Education, University of Ottawa; 4 Department of Surgery, Post-Graduate Programme, University of Guyana, Guyana; 5 Program for Educational Research and Development, Faculty of Health Sciences, McMaster University, Ontario, Canada; 6 Department of Family Medicine, Faculty of Health Sciences, McMaster University, Ontario, Canada

## Abstract

**Background:**

Eyal and Hurst proposed that locally relevant medical education can offset the prevalence of physician “brain drain” in resource-poor regions of the world, and presented a framework of the ethical and pragmatic benefits and concerns posed by these initiatives. The present study explored the framework’s utility through a case study of the University of Guyana Diploma in Surgery (UGDS) program

**Methods:**

The framework’s utility was evaluated using a case study design that included review and analysis of documents and semi-structured interviews with graduates, trainees, faculty members, and policy makers associated with the UGDS program. Data were analyzed from constructivist and interpretivist perspectives, and compared against the benefits and concerns described by Eyal and Hurst.

**Results:**

The framework is a useful template for capturing the breadth of experience of locally relevant training in the Guyanese setting. However, the results suggest that delineating the framework factors as either beneficial or concerning may constrict its applicability. The case study design also provided specific insights about the UGDS program, which indicate that the Program has promoted the retention of graduates and a sustainable culture of postgraduate medical education in Guyana.

**Conclusion:**

It is suggested that the framework be modified so as to represent the benefits and concerns of locally relevant training along a continuum of advantage. These approaches may help us understand retention within a resource-poor country, but also within particularly remote areas and public health care systems generally.

## Background

Surgical care is a critical component of effective health care systems. Sadly, there are significant disparities in surgical care worldwide, with enormous deficits in resource-poor countries,^1^–^5^ which contribute to diminishing economic productivity and quality of life, and the burden of poor health in already disadvantaged populations.^6^ Global surgery initiatives in these countries remain challenged by poorly-maintained facilities, inadequate access to medicines, supplies, and equipment, and, perhaps most importantly, a shortage of human health resources.^6^,^7^ Accordingly, the World Health Organization advocates for a scaling-up of health professions education in low-and middle-income countries.^8^–^11^ Through training, the distribution of health professionals can be balanced across regions in a way that promotes the provision of effective, safe, quality health interventions, which, in turn, will improve health outcomes and health systems.

To date, the highest impact education programs for increasing surgical capacity have been based on successful partnerships with academic institutions in high-income countries.^12^–^20^ Additionally, creating training opportunities that are based in resource-poor settings is key. Increasing the number of health workers in resource-poor countries is hampered by their eventual migration to high-income nations, often in pursuit of training opportunities that are otherwise inaccessible.^21^–^23^ Retention represents an important return on investment given that the loss of health professionals places further strains on countries and institutions that have invested limited resources into training. As such, health systems create incentives to influence physicians’ retention, including increased salaries and/or the provision of housing benefits. Similarly, disincentives may include recompensation demands from departing professionals or compulsory service bonds.^24^

Medical education is most effective at promoting retention when it is locally relevant: accountable to the local needs, understanding of the local environment, and empowering of local actors.^13^ In this regard, Eyal and Hurst^22^ have advocated for educational reforms that ensure that training focuses on local diseases, adapts to the available resources, and sets rotations in the high priority retention areas (i.e., rural regions). They developed a framework for retention based on their critical reflection of locally relevant training programs. The framework outlines eleven factors –five benefits and six concerns – that should be considered when implementing and/or evaluating locally relevant training. The five benefits include the promotion of contextually relevant skills, alignment with actual practice, the prestige of local professional practice, the recruitment of learners from high priority retention areas, and the development of local career options. The framework’s six concerns include the potential for inadequate training, breach of freedom of education and occupation, breach of freedom of movement, the creation of new inequalities, the hypocrisy of high-income partners, and a lack of support from key stakeholders (Table 1).

It is our position that the Eyal & Hurst framework may serve as a useful approach to evaluate global educational programs aimed at improving retention and building local capacity. In this study, the utility of this framework as a tool for evaluating program retention is examined with respect to its utility and comprehensiveness in explaining the context of the University of Guyana Diploma in Surgery (UGDS). This is a postgraduate surgery program in which the Canadian Association of General Surgeons (CAGS) has paired with the University of Guyana to provide a locally relevant program aimed at improving national health outcomes, retaining graduates, and building a sustainable local culture of graduate surgery education in the rural Guyanese context.

In conducting a case study examination of the impact of the UGDS Program on the retention of surgical trainees, our intentions were two-fold. First, we were interested in testing the general utility of the Eyal and Hurst’s framework. Thus, we analyzed the resultant data with specific respect to the degree to which it could be accounted for within the framework’s stated factors of benefit and concern. In doing so, we were afforded the opportunity to understand the comprehensiveness and appropriateness of the framework in a specific context. Secondly, the case study approach also served as a form of program evaluation for the UGDS Program, at least with respect to its impact on graduate retention. Thus, we also present a summary of the evaluation findings and recommendations for Program improvement.

## Methods

### Design

In order to investigate the utility of the Eyal and Hurst framework, the UGDS participants’ lived experiences were examined by way of in-depth qualitative interviews and document review. This constituted a case study design^28^ grounded in the constructivist^29^ and interpretive^30^ perspectives.

### Context

Guyana is a lower-middle income country in the Latin America and Caribbean region. While communicable diseases present challenges to the health status of the country’s population, the focus of health policy and resource allocation is increasingly shifting towards non-communicable diseases, which account for Guyana’s highest burden of mortality and morbidity. Priority areas include reducing the physiological consequences and mortality associated with accidents, injuries, and violence, and reducing pregnancy related complications,^25^ all of which can be improved through enhanced surgical training.

The delivery of health services in Guyana is provided at five levels, accessible through an upward referral system. At the fourth level of the health tier are the four Regional Hospitals, located in the rural towns of Linden, New Amsterdam, West Demerara, and Suddie. These hospitals provide emergency services, routine surgery, dental and diagnostic services, and obstetrical and gynecological care. When complications arise with patients or advanced surgeries are necessary, patients are transferred to the highest level of care – the National Referral Hospital - Georgetown Public Hospital Corporation (GPHC), in Georgetown, the country’s capital and largest urban centre. In contrast to the Regional Hospitals, GPHC provides a larger variety of diagnostic and specialist services. As well, the nature of practice differs in that surgeons at GPHC have a significantly heavier caseload, with more complex and diverse cases.

For many years, there were no postgraduate training programs in Guyana. Those interested in practicing surgery left the country for an overseas qualification, with very few returning to Guyana.^26^ In 2006, Guyana’s first surgical postgraduate training program was established through a strong partnership with the CAGS.^26^,^27^ The two-and-a-half-year program involves clinical rotations, structured tutorial modules, written and oral exams, and a six-month work placement in a rural-based Regional Hospital. The remainder of teaching is based out of Georgetown Public Hospital Corporation

To foster retention, trainees are provided housing benefits, and graduates of the program have a required return-of-service to the Ministry of Health (4 years). To date, the UGDS program appears to have played a key role reducing the number of emigrating medical professionals - 11 of the program’s 14 resident graduates now practice in Guyana – yet it has not been subjected to formal evaluation. In this regard, a program evaluation of the UGDS’s impact on retention for future professional practice in Guyana and within the program serves as a useful lens for an exploration and critique of Eyal and Hurst’s framework.

### Participants

Purposeful sampling^31^ was used to select trainee, graduate, faculty, and policy-maker participants for personal interviews. Twenty-six individuals with ties to the UGDS program were approached for interviews. Initially, 18 individuals responded, but two withdrew their participation due to scheduling conflicts. In total, interviews were conducted with eight graduates (eight men, mean age = 36 years), two trainees (two men, mean age = 30 years), four faculty members (two men, two women, mean age = 44 years), and two policy makers (one man, one woman, mean age = 51 years). All participants provided informed consent prior to participating according to the guidelines set out by the University of Guyana’s research policies and the Declaration of Helsinki (2013).

### Interviews

A script for semi-structured interviews was developed to explore the experiences and perspectives that may influence Program participants’ decisions to remain in Guyana; specifically within the public sector. These questions were written with consideration for locally relevant training, and the various incentives and disincentives designed to promote graduate retention and/or mitigate migration. Importantly, in generating these questions, the research team ensured that they were appropriately reflective of the factors of benefit and concern highlighted by Eyal and Hurst (2008) (see Table 1). The interview script was refined through pilot testing at the 2014 Bethune Roundtable Conference, an international surgical conference that discusses challenges and solutions to improving surgical care to under-serviced and marginalized populations in low- and middle-income countries.

Faculty and graduate participants were asked to describe their current professional roles, surgical career paths, intended involvement with the program, and strategies for strengthening career paths for future trainees. Specific questions on gender, age, hometown, marital status, family composition, educational attainment, and places in Guyana that the graduates have worked, trained, and lived previously were also incorporated. Interviews were conducted in English, Guyana’s official language. The lead researcher (AP) conducted interviews from June to November 2014. All interviews were audio-recorded and transcribed. Transcripts were checked for accuracy against the recordings.

### Reviewed documents

Reflective journals and presentations that were completed as assignments by participant learners following their rural training were reviewed for content pertaining to the framework.

### Data analysis

We sought to contextualize the data within the framework for locally relevant training. We used a combination of template and inductive analyses,^32^ which commenced by reading the dataset for content pertaining to relevant aspects of the framework. Two researchers used the constant comparative method^33^ over iterative stages of coding to refine the data into units. The units were then sorted into categories that reflected the eleven framework factors. Additional categories were created for segments of data that described a new theme not described by the framework. The final confirmation of categories and themes occurred after several rounds of coding and following discussion and resolution of divergent interpretations with the research team. All categories were mutually exclusive so that each unit of data was placed into only one category.^33^ The data collection stopped when the categories and new data gathering ceased to generate new insights.^34^ In doing so, the comprehensiveness to which the framework accounted for the coded data served as an index of the framework’s utility. To facilitate this appraisal, we recorded the numerical count of the units mapped to each of the relevant components. Throughout the analysis, we ensured qualitative rigor by both employing member-checking through respondent feedback and establishing an audit trail of the analytical process. NVivo 9.2 software was used to manage the data.

## Results

The analysis identified 938 units, 843 of which aligned directly with Eyal and Hurst’s (2008) framework for locally relevant training. Specifically, the analysis confirmed existing categories and the remaining 95 units did not coalesce under a new category or categories that differed from those outlined in the framework. However, certain concepts emerged as subcategories under the main themes. We describe these results below with illustrative excerpts from the dataset.

### Benefits

#### Context-relevant skills

There were 52 units that reflected this factor, indicated overwhelmingly positive views that the UGDS program teaches skills relevant to the Guyanese demographic and low-resource settings. The training program was frequently described as basic, but sufficient for meeting the needs of the community.

*The program doesn’t teach you… advanced techniques. [It is] Basic surgical training to survive in the community. The routine stuff. Appendicitis, hernias, trauma.* (Graduate, G01)

The relevance of the training was perceived as extending beyond surgical skills to also encompass understanding of the rural resources and protocols, which may represent an overlooked but very critical component of many locally relevant training models. Learners also commented on their confidence and preparedness for working in a resource-limited setting as a result of the program.

*It’s not just in this case, the surgical craft but it’s a lot of it is how to deal with the resources you have and how you use those resources. Paperwork. And everything that goes - the protocols in the region.* (G03)

Of the eight graduates interviewed, three had fellowship experience abroad, which demonstrates that the skills acquired may be transferable to other settings. As Eyal and Hurst suggest, contextually appropriate knowledge will make physicians maximally helpful in the regions they work. However, their claim that contextually appropriate knowledge makes graduates’ skills *less* relevant for work elsewhere is questionable. As seen in the Guyanese situation, contextually appropriate knowledge is insufficient to stem brain drain.

#### Alignment with actual practice

The data coded under this factor (50 units) indicated that there were varying levels of practice-associated burnout among the participants. Participants responded positively, indicating satisfaction with their role, positive working relationships, and feelings of appreciation, but also expressed significant frustration. However, this frustration was not attributed to a lack of preparedness, as is suggested by the framework. Rather, participants acknowledged a sense of frustration being overworked, understaffed, and having a high degree of responsibility.

*All the responsibility was held on our shoulders* and *sometimes it can feel overwhelming.* (G07)

*They [the trainees] feel like they are doing most of the work.* (Faculty, F01)

Review of the data suggests that despite alignment with actual practice, the locally relevant training did not reduce trainee burnout. It is important to consider the immense responsibility that will be placed on individuals that graduate from locally relevant training programs, particularly in regions lacking trained specialists. Thus, the framework should consider the possibility that burnout may increase with the implementation of locally relevant training, at least until adequate capacity has been built. To assume otherwise is overly naïve.

#### Enhanced prestige of local practice

This factor was reflected in 65 data units. Multiple participants commented that graduates are respected and perceived as competent surgeons by their peers and among patients. There was consensus however that the program’s prestige is compromised by the qualification that is ultimately granted. Since the inception of the surgery program in 2006, six other non-surgical postgraduate training programs have been implemented in Guyana that provide a higher qualification (i.e., Master’s degree). As a result, the prestige of the surgical program has diminished over time.

*The UGDS program is perceived to be one of the weakest postgrad programs. Not necessarily because of its content. But because of the outcome measure, which is the Diploma*. (G02)

Importantly, the role of the CAGS influenced the perceived prestige of the program positively.

*Their [CAGS] visit was helpful both from a technical point of view but more importantly provided credibility for my work.* (G01)

Positive perceptions of prestige will undoubtedly improve recruitment into the Program and in turn, retention. This is integral for building capacity and program sustainability. However, the results suggest that these impacts may be jeopardized by the view that postgraduate programs that award higher qualifications are more respected.

#### Local recruitment

Two trainees interviewed were from a rural hometown, while the others identified Georgetown as their hometown. A total of 192 units were identified within this category. The data were captured under two subcategories: perceptions of rural practice and experiences during rural training.

With respect to perceptions of rural practice, *g*raduates reported that the rural training improved their understanding of the challenges faced in under-resourced areas. As a result, graduates reflected that they are more inclined to practice in the rural setting and indicated a strong desire to “give back” and “make a difference.” However, participants also noted that the Rural Centres referred cases to Georgetown routinely, which was perceived as a limitation to the learning experience:

*So that was a challenge. The patient load [in rural setting] I didn’t believe was sufficient enough for my training experience, to improve at the time that I did it.* (G04)

Other graduates commented on these attitudes, views and practices as being entrenched, and the subsequent difficulty fostering positive change in these rural areas. A disconnect between the learning objectives of the trainees and the goals and attitudes among those in the Regional Hospitals may impede the potential for capacity building and development through locally relevant training in the regions.

With respect to the learning experience during rural training, a recurring theme among graduates, trainees, and faculty was a compromised learning environment, the interaction of limited work availability, a lack of support from rural staff, and resource deficits. Many participants explained that they were less inclined to return to the rural setting as a result.

*It [the rural experience] was adversely affected by frequent power outages and water shortage plaguing the hospital...* (G09)

In contrast, several graduates and trainees commented that the rural learning offered a unique opportunity to gain experience practicing surgery independently. The trainees were required to make decisions and lead administrative aspects of surgical care, which were perceived largely as challenging but valuable experience.

*It [being in rural setting] gave you a chance to be a bit more responsible. With regards to your previous work here [Georgetown Public Hospital Corporation] you always have the consultants and senior people here to fall back on.* (G03)

The trainee and graduate respondents also commented that the rural training component contributed to their personal maturation. It was suggested that the experience “*built character.*”

*The time spent in [the Regional hospital] was challenging but I grew as a person and matured as a surgeon-in-training.* (G01)

Participants acknowledged the importance of keeping trainees in these rural settings engaged through professional development. Generally, participants considered the public sector a better learning environment than the private sector because of the diversity of cases and the opportunities to engage with other students. Providing opportunities for professional development may help to address these limitations and foster a good learning environment that promotes retention.

Overall, the data units in this category suggest that rotations and recruitment from under-resourced regions may both contribute positively and negatively to retention in the rural setting. With respect to Eyal and Hurst’s framework, it may be overly idealistic to assume trainees will be content to remain in the rural setting. It is clear that to many, the rural setting is perceived as a temporary post, rather than a final destination. Assuming individuals may wish to pursue additional training, there is a need for integration with models of locally relevant training. This may involve the development of fellowship programs in surgical subspecialties that are feasible for graduates to pursue following completion of their general surgery training.

#### Improved local career options

This theme resonated strongly in the dataset, with 181 units aligning under this factor. Participants reflected that involvement in the program contributed to promotion and the ability to obtain more senior positions (for e.g., Senior Registrar).

*Retention is being driven by the fact that… they have graduated from the program [and] they are being absorbed by higher levels within the hierarchy of service.* (F03)

Participants agreed that they were frustrated by what they perceived as uncertain career paths and cited the need for more formalized career advancement. Again, several participants explained that the UGDS program grants a Diploma, which is insufficient for those seeking to practice at a higher level, as consultants.

*You’ve taken them [the trainees] to a certain level then what? So these guys are thinking I want to go ahead.* (F01)

## Concerns

### Poor quality of care

The participants commented that rural training improves the availability of surgical services, fosters a culture of evidence-based practice, and is ultimately beneficial to the quality of care in the region (42 units). In general, the perception is that basic enhancements in work conditions will play a significant role improving the quality of care irrespective of any changes in the UGDS program. In turn, this will have implications for retention because graduates and trainees prefer to be at the “centre of excellence” and are dissatisfied remaining in a setting where they perceive the quality of care to be sub-standard.

### Breach of freedom of education and/or occupation

The analysis elicited 49 data units related to this factor. Participants acknowledged going elsewhere to pursue further training as a potential option, including study in Jamaica, Trinidad, North America or the United Kingdom. However, participants opted to study in Guyana suggesting it was the more feasible option.

*Personally, UG was my best option. The other guys from our year that actually went to the University of the West Indies and it’s a little more difficult to get into that program because it’s a paid program – you actually have to pay to get it done. You have to get a job. You have to write their exams to get in. And then they usually take their local people first and if there are spots then they give the foreign people. So I wasn’t really prepared to go through all of that, being re-located and everything.* (G07)

Participants did not specifically indicate that they felt they were coerced to study in Guyana. For the most part, the program does not deny educational options to learners. Many participants outlined intentions to pursue additional training elsewhere. Freedom of education may be challenged among graduates fulfilling their return of service period, since they may not be permitted to leave the country during this time. Eyal and Hurst also raise a concern that locally relevant training may breach the freedom of occupation by deliberately limiting career options available to the graduates of these programs. This was expressed among several participants who felt that the qualification they received limited their opportunities for career advancement. This was often a key motivation for participants to seek training abroad.

#### Breach freedom of movement

There were 12 units coded under this factor. The results suggest that the return-of-service period for graduates of the program were perceived negatively, but only relative to the formal exit restrictions of other postgraduate programs in Guyana. One participant summarized the key concerns with the formal exit restriction:

*It’s something like, five or seven years of contract, when the program is just two-and-a-half years. And at the end of the program, you just have a Diploma; it’s not a good trade-off. Because there are other programs now, that are four years, and at the end of it you’re given an MMed. And it’s the same exact contract in terms of time.* (G08)

Lengthy formal exit restrictions may deter prospective learners from joining the program and this may adversely impact capacity building within the program.

#### New inequalities

Participants perceived unequal opportunity relative to graduates of other postgraduate programs (19 units). In particular, the respondents once again pointed to the qualifications granted by other programs as preferential to employers.

*Because it’s a Diploma program, it’s never complete…You have to compete with other surgeons who have far higher qualifications than you. It doesn’t necessarily mean they can do more than you or that they can do it better. It’s just the way things are*. (G07)

In this view, one may consider the Diploma qualification as perpetuating unequal opportunities. As well, given that the program only has male graduates thus far, this may represent an area of unequal opportunity, in which women face unique barriers to accessing the training program compared to their male counterparts. Eyal and Hurst claim that unequal opportunities among students may arise due to differences in socioeconomic status. That is, locally relevant training that is subsidized may be the only feasible option for lower income learners, while higher income learners can pursue training abroad. However, this was not reflected in the data.

#### Partner hypocrisy

There were 32 units reflecting this theme in the dataset. When asked about the role of Western institutions relative to the UGDS program, participants responded overwhelmingly that the CAGS involvement in Guyana was not hypocritical in this fashion. Participants spoke of the CAGS partnership positively, as collaborative and built on “friendship, respect, and mutual trust.”

I think it [the role of CAGS] is supportive. I think we benefit from when faculty comes down, when fellows come down, when junior staff, when they come down they share their perspective and knowledge and so on. I think the guys who go on these clinical fellowships or observerships, they benefit from being exposed to what it kind of should be like. I don’t think any of them have left because of that exposure. So I don’t think it really contributes [to physician migration]. (F02)

In review of the data it seems unlikely that the CAGS involvement contributes to brain drain in the Guyanese context. In this regard, the framework should account for the positive contributions of globally-accountable institutions from high-income countries, which include mentorship, prestige, and shared knowledge.

#### Lack of support for the reform

Participants identified faculty members, senior staff, fellow learners, the Institute of Health Science Education, (Georgetown, Guyana), Georgetown Public Hospital Corporation, the University of Guyana, and the Ministry of Health (Guyana) as key stakeholders in the UGDS program. The data analysis revealed 149 units in which participants spoke of support, or a lack thereof, from these key players. Participants spoke positively about the helpful role faculty and staff played in supporting learners, describing them as “readily available,” “willing to be consulted at any time,” and “encouraging and accessible during times of need.” A resounding theme, however, was the support that the learners themselves provided to the UGDS program. Overall the graduates and trainees indicated a strong willingness and commitment to building capacity as Program educators and leaders in the future.

*I see myself as wanting to play a pivotal role in advancing the program and continuing what’s already been started*. (G02)

There is very clear evidence that the UGDS program has the support of its trainees, graduates and faculty members, and that they welcome the opportunity to contribute to improved program capacity.

## Evaluation summary

The case study approach yielded important insights about the UGDS program itself, which represent an important secondary outcome of this work. Specifically, the data yielded insights about the influence of Guyana’s surgical training program on the retention of its graduates in the Guyanese health sector as well as within the Program. Overall, the analyses suggest that the Program’s impact is positive in both regards. The majority of the graduates interviewed are currently practicing in Guyana. Importantly, all interviewees stated a strong willingness to engage with the Program as educators and developers of further specialist education in Guyana.

In summary, the results imply that the Program’s rural training component raises awareness about the complexities of providing surgical care in under-resourced areas while also providing learners with an opportunity to practice independently and assume leadership positions. In doing so, the Program is effectively preparing learners with the skills and knowledge to function in resource-limited Guyanese settings. This is viewed as influencing retention positively. However, there are also areas where focused efforts may further enhance retention; particularly, in the public sector and rural regions. According to participants, there were a number of influences that may contribute to future decisions to migrate from the Regional Hospitals. These can be best summarized in terms of the learners’ frustrations with both practice and educational experiences in these settings. In particular, a lack of local support, inadequate facilities, and poor organizational attitudes compromised learning and were cited as areas for improvement.

Ultimately, one’s choice to leave the Guyana health system altogether, although rare, was most often attributed to a perceived desire for advanced training and qualifications that would support opportunities for career growth. It is recommended that the Program address these perceived areas where possible. For instance, formal exit restrictions may be revised so as to compare with those imposed by other national post-graduate programs. Perhaps most saliently, the ultimate qualification that is granted by the program may be reconsidered. It was a widely held view among students that the diploma distinction leads to unequal job opportunities as compared to other potential degrees (for e.g., Masters).

## Discussion

The UGDS Program served as a useful case study for a deeper exploration of Eyal and Hurst’s framework. In this regard, the data demonstrate the framework’s comprehensiveness and utility. Specifically, related themes emerged: improved career options, support for the reform, and the contextual importance of rural training. Nevertheless, the data also indicate the framework could be refined to reflect more accurately the impact of each factor on migration and/or retention. For instance, Eyal and Hurst delineate between the potential benefits and concerns of locally relevant training. However, the study results suggest that the framework factors are better understood along a continuum rather than a dichotomy.

The need to consider the framework factors along a continuum from benefit to detriment is apparent when one reviews the data associated with context-relevant skills and alignment with actual practice. However, the need to think of a continuum rather than a dichotomy is clearly illustrated in their claim that involving partner institutions can promote counter-productive recruitment practices. We discovered, to the contrary, that the CAGS’s involvement was perceived to be focused resolutely on building local capacity. This is encouraging for the Canadian partners insofar that it instills confidence that they are contributing to the overall goals of the program. The reciprocity of learning that occurs through such a program together with the personal rewards that accompany this socially accountable practice, demonstrate that the endeavor was largely positive and worthwhile. We understand that our findings do not preclude the possibility that in different circumstances the involvement of partners could be self-serving but we instead point out that such partnerships have the potential to provide benefits. Taken together, our recommendation is that the factors in the Eyal and Hurst framework be revised so that each factor be viewed along a range from beneficial to problematic.

The success of this framework in describing the impact of locally relevant training on retention in the Guyanese context leaves us hopeful that it may be similarly useful in other resource-poor regions. In particular, we are optimistic that it can provide insights within each of three types of retention: a resource-poor country, rural/remote areas, and public health systems. By using Eyal and Hurst’s factors to analyze each of these three types of retention, programs will be equipped to leverage training advantages to offset the out-migration of health professionals.

The Eyal and Hurst framework may also be relevant for much of the training that is conducted in the Canadian setting, which includes rural or remote areas and regions with a high prevalence of marginalized or culturally-diverse patient populations. For example, a sustained shortage of physicians in Northern Ontario led to the development of the Northern Ontario School of Medicine, which aimed to establish rural-based education that recruits and retains students from the local region.^35^ This school and others like it (e.g., Dalhousie University, McMaster University, University of Northern British Columbia) also rely on focused recruitment, rural experiences, and locally relevant curricula to foster positive perceptions and the goal of a more adequately distributed health work force.^36^ The comprehensive nature of Eyal and Hurst’s framework for locally relevant training provides these institutions with a clear lens with which to evaluate their programs and initiatives.

In closing, we recall Hodges’s warning that “the dominance of particular countries or regions is almost certain to lead to the marginalization of priorities, values, content knowledge, and exposure to learning contexts of less dominant countries or regions.”^37^ We are reminded that vast differences between contexts present unique challenges to training health professionals that are well-positioned to meet the needs of the host population. Our findings show that locally relevant training can in fact foster retention in rural and remote settings of low income countries.

## Figures and Tables

**Table 1:** Eyal and Hurst’s (2008) Framework for Locally Relevant Training

Benefits	Concerns

**Context-relevant skills**	**Inadequate training**
Locally relevant training teaches skills that are more relevant for work in under-resourced areas and less relevant for work in high resource areas (i.e., the private sector; high income countries).	If the standard of locally relevant training is inadequate, then poor quality of care in the region may result.
**Alignment with actual practice**	**Breach of freedom of education and/or occupation**
Locally relevant training reduces the discrepancy between the expectations of education and the reality met on the ground and, in turn, frustration and burn-out	Locally relevant training may coercively prevent students from pursuing alternative training options.
**Enhanced prestige of local practice**	**Breach of freedom of movement**
Locally relevant training raises the prestige of local jobs.	Formal exit restrictions associated with locally relevant training may violate an individual’s inherent freedom of movement.
**Local recruitment**	**New inequalities**
Locally relevant training in resource-poor regions fosters local recruitment and retention.	Locally relevant training programs may inadvertently create unequal opportunities between lower-income students constrained to local options and higher-income learners afforded more choice.
**Improved local career options**	**Partner hypocrisy**
Locally relevant training results in improved local career options.	High-income partner institutions may be hypocritical by advocating for locally relevant training while also engaging in harmful recruitment practices.
	**Lack of support for the reform**
	Local stakeholders may view locally relevant training as a threat to professional prestige, the quality of care, or their own careers.
